# O-38 - HUMAN MYCOBACTERIUM BOVIS TUBERCULOSIS IN NORTHERN IRELAND: A TEN-YEAR RETROSPECTIVE REVIEW OF EPIDEMIOLOGY, EXPOSURES AND OUTCOMES

**DOI:** 10.1093/pubmed/fdag046.034

**Published:** 2026-07-09

**Authors:** Dr Aoife Nic Iomhair, Kate Jackson, Louise McCorry, Timothy Shaw, Derek Fairley, Jillian Johnston, Lynsey Patterson, Suzanne Wilton, Mark O' Doherty, Siobhan Lavery

**Affiliations:** Public Health Agency; Belfast Health and Social Care Trust; Belfast Health and Social Care Trust; Belfast Health and Social Care Trust; Public Health Agency; Public Health Agency; Public Health Agency; Public Health Agency; Public Health Agency; Public Health Agency

## Abstract

**Background:**

Since 2019, an apparent increase in human *Mycobacterium bovis* tuberculosis (TB) notifications has been observed in Northern Ireland (NI), coinciding with increasing prevalence of bovine TB in cattle. Clinical concerns have also been raised regarding severe disease and atypical epidemiological profiles.

**Objectives:**

To evaluate temporal trends in human *M. bovis* TB in NI and across the United Kingdom (UK); characterise case demographics and exposure histories; describe clinical outcomes and identify factors associated with adverse outcomes.

**Methods:**

A retrospective review of *M. bovis* TB cases in NI between 2013 and 2023 was undertaken. UK Health Security Agency data were used to compare incidence rates and the proportion of TB attributable to *M. bovis* across the UK. Local enhanced surveillance data described demographics and exposures. Clinical records were reviewed to characterise presentation, treatment and outcomes at two years. Associations with adverse outcomes were explored.

**Results:**

39 cases were identified. NI demonstrated higher incidence and higher proportion of TB attributable to *M. bovis* than other UK regions, with an increase observed since 2019. Most cases were UK-born, aged over 45 and resident in rural or mixed areas. 22 cases had identified exposures, most commonly occupational animal contact and unpasteurised milk consumption, while one third reported no identifiable exposure. Two thirds had pulmonary or lymph-node disease, and 38.5% were immunosuppressed. At two years, 43.6% experienced complications or death. Females and immunosuppressed patients had poorer outcomes.

**Conclusions:**

Human *M. bovis* TB remains uncommon but represents a higher and potentially increasing burden in NI. Most cases aligned with recognised epidemiological profiles, but some lacked typical risk factors. Findings support the need for strengthened surveillance and increased clinical and occupational awareness.

**Word Count:**

275

